# The yield of stress perfusion CMR in asymptomatic diabetics

**DOI:** 10.1186/1532-429X-13-S1-O70

**Published:** 2011-02-02

**Authors:** Darach O h-Ici, Thomas Hovasse, Marie Claude Morice, Yves Louvard, Thierry Unterseeh, Jerome Garot

**Affiliations:** 1 Institut Cardiovasculaire Paris Sud, Hopital Prive jacques Cartier, Massy, France

## Background

The benefit of screening asymptomatic diabetic patients for CAD remains unclear. A recent ADA consensus statement on this issue concluded that routine screening is not recommended. The aim of this study was to assess the yield of stress perfusion CMR in a diabetic population.

## Methods

All patients who underwent stress CMR at ICPS (tests, n = 4589) between November 2009 and September 2010 were identified through the use of the cardiology database. Exclusion criteria included (1) history of documented myocardial infarction (n = 623); (2) prior percutaneous coronary intervention (PCI) (n = 1335); and (3) prior coronary artery bypass grafting (CABG) (n = 264). For patients who underwent multiple tests during this time period (n = 116), only the first test was included. The study population consisted of 2737 patients.

## Results

Similar percentages of diabetic and nondiabetic patients had abnormal scans (34.8% vs 33.4%, *P* =0.48). However diabetic patients were more likely to have inducible ischemia than nondiabetic patients (14.4% vs 11.2%, *P* =0.02), whereas they were equally likely to have evidence of infarction on delayed enhancement images (23.7% vs 25.4%).

Asymptomatic and symptomatic diabetic patients had similar percentages of ischemia (13.5% vs 15.7%, p = 0.40), and infarction (24.9% vs 21.9%, p = 0.36).

With regards to symptomatic patients, patients with diabetes had significantly higher percentages of ischemia (15.8% vs 11.1%, p = 0.03), but similar amounts of infarction (21.9% vs 24.2%, p = 0.42) than symptomatic nondiabetic patients.

Ejection fractions after stress testing were similar in both groups (diabetic vs nondiabetic - 68.47% vs 69.11% p = ns).

## Conclusion

Stress testing in asymptomatic diabetics identifies a high percentage of patients with unknown myocardial infarction. This information has important prognostic implications. Asymptomatic diabetic patients have a significantly higher amount of ischemia than non-diabetic patients. Ischemia is the main predictor of adverse cardiac outcomes. Early identification of this can guide revascularization, which has been shown to improve prognosis in those with at least moderate ischemia.

